# Gut Microbiome Profiling Uncovers a Lower Abundance of *Butyricicoccus* in Advanced Stages of Chronic Kidney Disease

**DOI:** 10.3390/jpm11111118

**Published:** 2021-10-29

**Authors:** Tessa Gryp, Karoline Faust, Wim Van Biesen, Geert R. B. Huys, Francis Verbeke, Marijn Speeckaert, Jeroen Raes, Mario Vaneechoutte, Marie Joossens, Griet Glorieux

**Affiliations:** 1 Department of Internal Medicine and Pediatrics, Nephrology Section, Ghent University Hospital, 9000 Ghent, Belgium; tessa.gryp@poulpharm.be (T.G.); wim.vanbiesen@ugent.be (W.V.B.); francis.verbeke@uzgent.be (F.V.); marijn.speeckaert@ugent.be (M.S.); 2 Department of Microbiology, Immunology and Transplantation, Rega Institute, KU Leuven, 3000 Leuven, Belgium; karoline.faust@kuleuven.be (K.F.); geert.huys@kuleuven.vib.be (G.R.B.H.); jeroen.raes@kuleuven.vib.be (J.R.); marie.joossens@ugent.be (M.J.); 3 Laboratory Bacteriology Research, Department of Diagnostic Sciences, Ghent University, 9000 Ghent, Belgium; 4 Center for Microbiology, VIB, 3001 Leuven, Belgium; mario.vaneechoutte@ugent.be; 5 Laboratory of Microbiology, Department of Biochemistry and Microbiology, Ghent University, 9000 Ghent, Belgium

**Keywords:** chronic kidney disease, uremic toxins, gut microbial composition, *p*-cresyl sulfate, *p*-cresyl glucuronide

## Abstract

Chronic kidney disease (CKD) is characterized by the accumulation of uremic toxins which exert deleterious effects on various organ systems. Several of these uremic toxins originate from the bacterial metabolization of aromatic amino acids in the colon. This study assessed whether the gut microbial composition varies among patients in different stages of CKD. Uremic metabolites were quantified by UPLC/fluorescence detection and microbial profiling by 16S rRNA amplicon sequencing. Gut microbial profiles of CKD patients were compared among stages 1–2, stage 3 and stages 4–5. Although a substantial inter-individual difference in abundance of the top 15 genera was observed, no significant difference was observed between groups. Bristol stool scale (BSS) correlated negatively with *p*-cresyl sulfate and hippuric acid levels, irrespective of the intake of laxatives. *Butyricicoccus*, a genus with butyrate-generating properties, was decreased in abundance in advanced stages of CKD compared to the earlier stages (*p* = 0.043). In conclusion, in this cross-sectional study no gradual differences in the gut microbial profile over the different stages of CKD were observed. However, the decrease in the abundance of *Butyricicoccus* genus with loss of kidney function stresses the need for more in-depth functional exploration of the gut microbiome in CKD patients not on dialysis.

## 1. Introduction

In chronic kidney disease (CKD), uremic toxins accumulate in the blood circulation [1,2,3], exerting deleterious effects on various organ systems of the human body [4] and contributing to cardiovascular morbidity and mortality [5,6,7,8,9]. The gut microbiota is responsible for the generation of the precursor metabolites of the protein-bound uremic toxins (PBUTs) such as *p*-cresyl sulfate (*p*CS), *p*-cresyl glucuronide (*p*CG), indoxyl sulfate (IxS) and indole-3-acetic acid (IAA). Aromatic amino acids (i.e., tyrosine, phenylalanine and tryptophan) are predominantly metabolized in the distal colon by gut bacteria into *p*-cresol, indole and IAA [10,11,12,13,14]. Subsequently, *p*-cresol and indole are detoxified through sulfation and glucuronidation by the colon mucosa and liver into *p*CS, *p*CG and IxS [15,16], whereas IAA enters the blood circulation unmodified [17,18]. In the circulation, these PBUTs reversibly bind to albumin [19]. Since only the free fraction can be removed by dialysis therapy [20,21], albumin-binding hampers their removal at end-stage kidney disease (ESKD).

As shown in previous studies [11,22,23,24,25,26,27,28], the abundance of specific gut bacteria in patients with CKD is altered. A previous study by our group using 16S rRNA amplicon sequencing [29] revealed that hemodialysis (HD) patients do not have a uniform altered gut microbial composition, when compared to healthy volunteers with similar genetic and environmental backgrounds from the Flemish Gut Flora Project (FGFP) [30]. However, when dividing the CKD patient group according to their serum uremic toxin levels into a high *p*CS and low IxS group and a high IxS and low *p*CS group, the bacterial genera *Enterococcus*, *Dialister*, *Akkermansia* and *Ruminococcus* were comparatively overrepresented in the high *p*CS/low IxS group, whereas members of *Bacteroides* and *Blautia* were comparatively overrepresented in the high IxS/low *p*CS group [29]. Nevertheless, because only patients who had reached ESKD were included in this study, it cannot be ruled out that dialysis-related conditions co–influenced microbiome readouts.

In the present study it was investigated whether the gut microbial profile changes over the different stage of CKD and whether gut microbial composition correlates to plasma levels of colon derived uremic toxins. The aim was to point out potential gut microbial targets to prevent the accumulation of uremic toxins from the early stages of CKD on, which could in its turn decrease morbidity and mortality of patients with CKD and/or slow down disease progression.

## 2. Materials and Methods

### 2.1. Study Population and Sample Collection

A total of 111 patients with CKD [CKD 1 (*n* = 13); CKD 2 (*n* = 23); CKD 3 (*n* = 44); CKD 4 (*n* = 22); CKD 5 (*n* = 9)] were included. The ‘Chronic kidney disease epidemiology collaboration (CKD-EPI)’-creatinine equation was used to determine the estimated glomerular filtration rate (eGFR) of each patient. Based on their eGFR, the total group of patients with CKD was divided into three groups: (i) eGFR above 60 mL/min/1.73 m^2^ corresponding to CKD stages 1 and 2 (*n* = 36), (ii) eGFR between 30 and 60 mL/min/1.73 m^2^ corresponding to CKD stage 3 (*n* = 44), and (iii) eGFR below 30 mL/min/1.73 m^2^ corresponding to CKD stages 4 and 5 (*n* = 31). From each patient of the CKD population, a single blood and fecal sample was collected in parallel. Bristol Stool Scale (BSS), a visual scale of the aspect of stool, from hard (1) to liquid (7) [31] was indicated by the lab technician immediately after obtaining the fresh stool sample from the patients. Patient characteristics and clinical parameters have been described previously [32]. 

Exclusion criteria were age < 18 years, active infection (C-reactive protein > 20 mg/L), active malignancy, cardiovascular event in the past three months, immunosuppressive therapy, inflammatory bowel disease, obesity (Body Mass Index > 35 kg/m^2^), pregnancy, transplantation, and/or use of non-steroidal anti-inflammatory drugs within the past month. Patients were asked to provide information on general diet and health status in a questionnaire including the occurrence of stomach, gut, liver and bile diseases, gastro-intestinal infection and antibiotic, pre- and probiotic and laxative intake and different types of surgery. All patients gave written informed consent before inclusion and the study was conducted following the Declaration of Helsinki, and approved by the Medical Ethics Committee of the Ghent University Hospital (Ref 2010/033, B67020107926).

### 2.2. Determination of Uremic Metabolites in Blood

Plasma urea [60 Dalton (Da)], creatinine (133 Da), phosphorus (31 Da) and total protein content were measured with standard laboratory methods in the routine laboratory of the Ghent University Hospital, Belgium. In plasma, total concentrations of the PBUTs *p*CS (187 Da), *p*CG (284 Da), IxS (213 Da), IAA (175 Da), 3-carboxy-4-methyl-5-propyl-2-furanpropionic acid (CMPF, 240 Da) and hippuric acid (HA, 179 Da) were measured by ultra-performance liquid chromatography (UPLC) and fluorescence detection as previously described [32,33].

### 2.3. Illumina-Based Microbial Profiling

Fecal DNA extraction using the RNeasy PowerMicrobiome Kit^®^ (Qiagen, Hilden, Germany) and Illumina-based microbial profiling were performed as described previously [29]. The V4 region of the 16S rRNA gene was amplified using the 515F/806R primer set. Sequencing data were analyzed using the DADA2 pipeline, filtering and trimming forward and reverse reads truncated after 130 and 200 bases. Thirty bases were removed from the start of forward and reverse reads. Minimum quality score of each read was >11 and reads with more than 2 expected errors (EE) were discarded. Identified chimeras were removed using removeBimeraDenovo. For taxonomic classification the 16S rRNA reference (RDP) training set, version 16, formatted for DADA2, revealed 4069 amplicon sequence variants (ASVs). Reads were rarefied to 21,046 reads per sample and ASVs that had no reads left after rarefaction were removed, resulting in 3574 ASVs (this number can vary slightly upon re-rarefaction). Metadata parameters for which more than half of the values were missing were discarded. Metadata, sequencing data and bacterial cell count data were obtained from a total of 111 subjects, for whom the metadata and bacterial cell count data were summarized in a previous paper including the same CKD cohort [32].

### 2.4. Statistical Analysis

Statistical analyses were performed with R. To identify, in an unsupervised manner, the main correlates of variation of the microbial composition in this cohort, two different variants of the same approach were applied: (i) a Principal Coordinates Analysis (PCoA) on rarefied data using Bray Curtis dissimilarities, and (ii) centered log-ratio (CLR) transformation in combination with Euclidean distances. PCoA was carried out on a sub-set of 102 samples without missing metadata values. Envfit was used to assess the co-variation of principal components with metadata in the triplot [34]. Differences between the gut microbial composition on genus levels between earlier CKD stages (CKD 1–2) and later stages of CKD (CKD 4–5) were assessed with ALDEx2 [35], with Wilcoxon rank sum test, *p*-values were Benjamini-Hochberg corrected.

## 3. Results

### 3.1. Gut Microbiome Profiles in Different Stages of CKD

**Figure 1:**
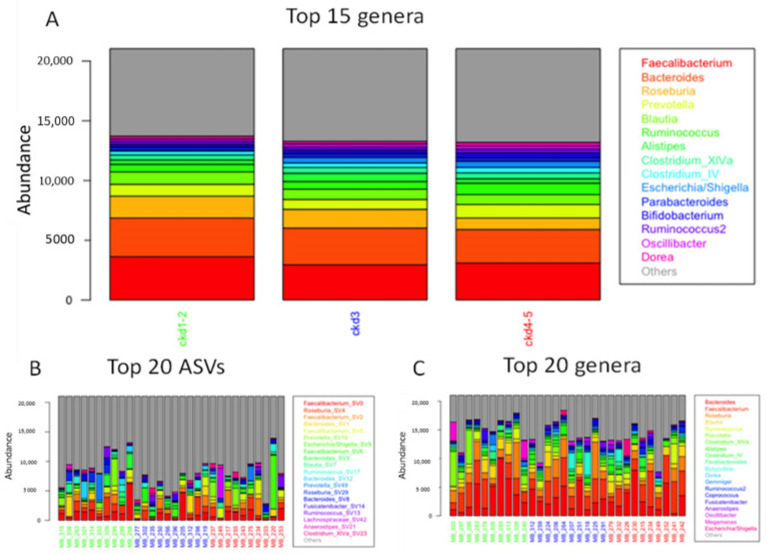
Mean abundance of the top genera or ASVs computed across all CKD stage-specific samples. (**A**) mean abundance of the top 15 genera in the different CKD stage groups; (**B**) mean abundance of the top 20 ASVs in 10 randomly selected samples per CKD stage group; (**C**) mean abundance of the top 20 genera in 10 randomly selected samples per CKD stage group. CKD: chronic kidney disease; ASV: 16S rRNA amplicon sequence variant.

**Figure 2:**
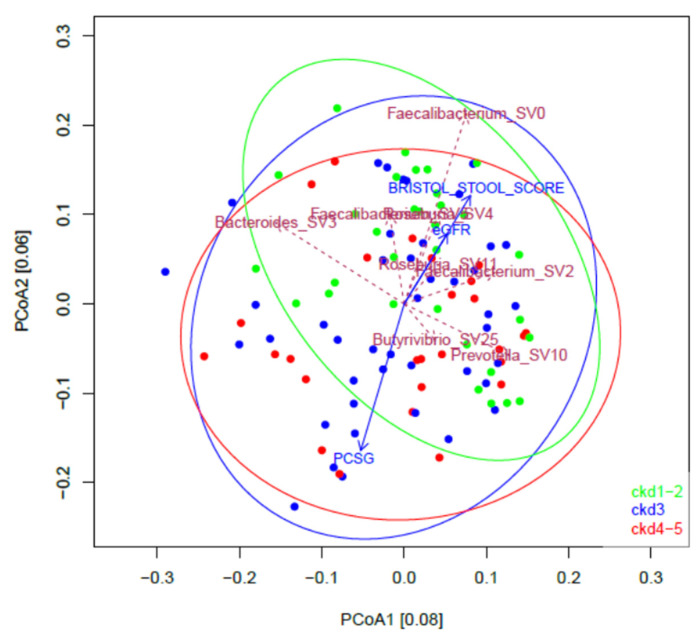
Principal coordinate analysis of the microbial composition of the fecal samples of patients with CKD. Colors and ellipses are coding for CKD stages; SV: (amplicon) sequencing variant; PCSG: *p*-cresyl sulfate + *p*-cresyl glucuronide; eGFR: estimated glomerular filtration rate.

Figure 1A illustrates the mean abundance of the top 15 genera in different groups of progressive CKD stages (CKD1–2, CKD3 and CKD4–5), computed across all CKD stage-specific samples and ranked across all samples. Across all groups, the three most abundant genera were *Faecalibacterium*, *Bacteroides* and *Roseburia*. No significant difference in genus abundances between the different CKD groups was observed. Nevertheless, inter-individual variation was apparent across abundance profiles of the top 20 ASVs (species level) and genera (sum of ASVs) of 30 randomly selected samples (10 per CKD group) (Figure 1B,C). Also based on PCoA (Figure 2), CKD clusters largely overlapped in ordination.

### 3.2. Correlates of Intestinal Microbiota Composition in CKD

A total of 3574 ASVs were found in the cohort. The missing-value-free metadata contained 62 parameters (Table S1), including toxin concentrations and confounders for microbiota research [29]. The (Bray-Curtis-based) variation of the microbial composition in this cohort was correlated to BSS, eGFR and *p*CSG, of which the latter is the sum of plasma *p*CS and *p*CG (Figure 2). The length of scaled arrows reflects the correlation with overall community composition. BSS and eGFR point in the same direction, and in the opposite direction as *p*CSG (Figure 2). This finding was confirmed by using Centred Log-Ratio (clr) transformation and Euclidean distances.

The correlation between plasma levels of intestinally generated uremic toxins and transit time was assessed by analyzing their correlation with BSS [1 (severe constipation) to 7 (severe diarrhea)]. A significant negative correlation was found between BSS and plasma HA in the total CKD cohort and in stages 1–2; and between BSS and *p*CS in the total CKD cohort and stages 4–5 (Table 1).

On the triplot in Figure 2, it is also apparent that *p*CSG points in the opposite direction as BSS. Because part of the samples were taken from participants on laxatives and to avoid distortion due to laxative usage, analyses were also repeated excluding the samples of patients on laxatives (*n* = 101). Similar significant correlations between BSS and *p*CS and HA were found (Table S2).

### 3.3. Variation in Gut Microbiota Profiles in Different Stages of CKD

**Figure 3:**
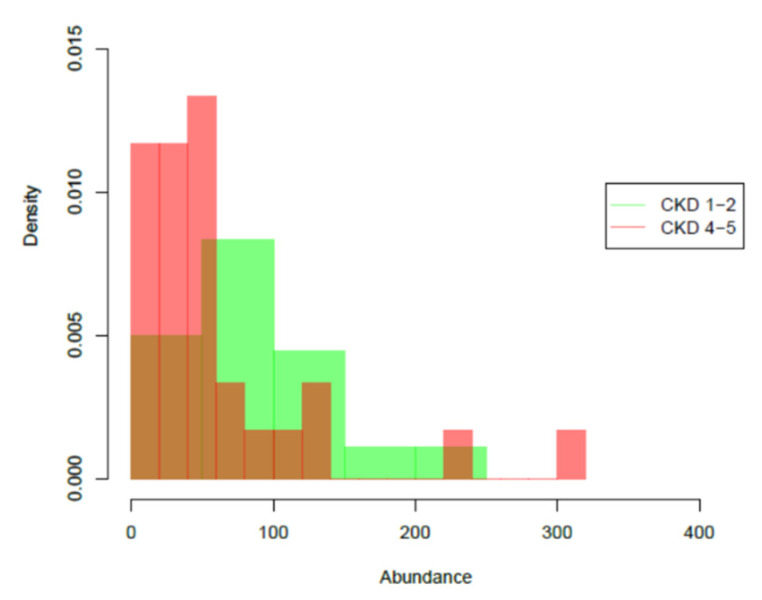
Significant differences of the abundance of *Butyricicoccus*, a genus with butyrate-generating properties, between earlier (CKD stages 1–2; green) and later (CKD stages 4–5; pink) stages of CKD. Brown: the intersection of both graphs. *p* = 0.043.

Potential microbial differences between the early stage and the most advanced stage CKD patients were assessed. No differences survived multiple testing correction using all 3574 ASVs nor using the 100 top-abundant sequencing variants. However, at genus level, a significantly lower abundance of *Butyricicoccus* was found in latest stages (CKD 4–5) of the disease compared to the earliest stages (CKD 1–2) (*p* = 0.043; Figure 3).

## 4. Discussion

In this study, the gut microbial composition was assessed among patients at different stages of CKD not on dialysis, i.e., CKD stages 1 to 5, taking their plasma levels of PBUTs into account. No specific microbial profile was linked to different degrees of kidney function decline, as was also observed in ESKD patients compared to the control group in our previous study [29]. However, at the genus level, a significantly lower abundance of *Butyricicoccus* was found in advanced stages of CKD compared to the earliest stages. This is in line with our previously reported quantitative polymerase chain reaction (qPCR) data showing that the abundance of *Butyricicoccus* spp. but also of other gut bacteria such as *Roseburia* spp., *Faecalibacterium prausnitzii*, and *Bifidobacterium* spp. declined with advancing stages of CKD [11] and confirm earlier qPCR findings in CKD by Jiang et al. for *Roseburia* spp. and *Faecalibacterium prausnitzii* [36]. In addition, a recent metagenome study showed that butyrate-producing species such as *Roseburia inulinivorans*, *Ruminococcus torques* and *Ruminococcus lactaris* were already less abundant in early CKD compared to the control group and this in the absence of clear gut microbiome changes [37]. Moreover, other studies in patients with ESKD also showed that butyrate-generating bacteria are reduced compared to controls [28,36,38,39] and that a decreased abundance of *Faecalibacterium prausnitzii* is also associated with inflammatory bowel disease (IBD) and irritable bowel syndrome [40,41,42]. These results justify further exploration of butyrate-producing gut bacteria for their modulatory potential in intestinal disorders [43].

The variation of the microbial composition in this cohort correlated to BSS, eGFR and the sum of *p*CS and *p*CG (Figure 2), which is in line with earlier findings in healthy cohorts (BSS, eGFR) [30] and also with our earlier findings for HD patients (BSS, *p*-cresyl conjugates) [29]. Similar to the HD cohort, BSS points in the opposite direction as the *p*-cresyl conjugates (*p*CS and *p*CG) (Figure 2). Interestingly, in the present CKD cohort, IxS was not identified as a main correlate of the intestinal microbial composition, although in the HD cohort, this was a main co-variate pointing in the same direction as BSS [29]. In addition, the overall negative correlation between BSS, as a marker of transit time, and plasma levels of HA and *p*CS, especially in a more advanced stage of CKD, suggests that it should be explored whether preservation/modulation of transit time could affect circulating levels of the *p*CS. Similar observations were made in patients on automatized peritoneal dialysis [44] and in in patients with non-dialysis-dependent CKD [45].

The lack of overt difference in gut microbiota composition between the different stages of CKD observed in the present study and in a previous study on HD patients by our group points to potential limitations of 16S rDNA-based approaches, and calls for more in-depth studies focusing on functional characteristics of gut microbiota. This is exemplified by the recent finding that dietary intake of sulfide donors can tune microbiota function via post-translational modification without altering microbial community composition [46]. Shotgun metagenomic approaches revealing relative abundances of gene pathways and metatranscriptomic studies in CKD cohorts are still in their infancy, but should be further explored to build a more comprehensive systems biology framework for the gut-kidney axis. 

The major strength of this study is that it covers the whole range of pre-dialysis CKD, while most studies focus on patients with ESKD. To follow up this cross-sectional approach, however, longitudinal sampling is preferred to also allow other covariates to be included in data analysis. In addition, given the high inter-individual variation observed, expansion of the cohort’s sample size could provide more power to reveal potential microbiota differences between subsequent CKD stages.

## 5. Conclusions

In this cross-sectional study we observed no gradual differences in the gut microbial composition in patients at different stages of CKD. However, a non-supervised comparison of CKD stage 1–2 with CKD 4–5 revealed a decrease in the abundance of the butyrate-producing genus *Butyricicoccus* with loss of kidney function. Additional in-depth studies taking into account the functional capacity of the gut microbiome will be required to identify potential targets to tackle chronic inflammation and to decrease levels of intestinally generated uremic toxins and their precursors.

## Figures and Tables

**Table 1:** Correlation between intestinally generated uremic toxins and transit time of patients with CKD.

Correlation to BSS	All CKD Stages (*n* = 111)		CKD Stage 1–2 (*n* = 37)		CKD Stage 3 (*n* = 44)		CKD Stage 4–5 (*n* = 33)	
Uremic Toxin	*r_s_*	*p*-Value	*r_s_*	*p*-Value	*r_s_*	*p*-Value	*r_s_*	*p*-Value
Indoxyl sulfate	−0.173	ns	−0.130	ns	−0.054	ns	−0.223	ns
Indole-3-acetic acid	−0.150	ns	−0.035	ns	−0.041	ns	−0.206	ns
Hippuric acid	−0.343	<0.001	−0.366	0.036	−0.153	ns	−0.318	ns
*p*-Cresyl sulfate	−0.287	0.003	−0.335	ns	−0.134	ns	−0.443	0.012
*p-*Cresyl glucuronide	−0.175	ns	−0.105	ns	−0.116	ns	−0.340	ns

BSS: Bristol stool scale which is used to assess transit time (slow transit = low BSS); CKD: chronic kidney disease; *r_s_*: Spearman’s correlation coefficient; bold: significant; ns: not significant.

## Data Availability

All data are fully available without restriction, in an anonymized format, upon request.

## Supplementary Materials

The following are available online at https://www.mdpi.com/article/10.3390/jpm11111118/s1, Table S1: Metadata (*n* = 62) taken into account for correlation to overall taxon composition.; Table S2: Correlation between intestinally generated uremic toxins and transit time of patients with CKD not on laxatives.
