# Supplementary material for: Gut Microbiome Profiling Uncovers a Lower Abundance of Butyricicoccus in Advanced Stages of Chronic Kidney Disease
Source: J Pers Med. 2021 Oct 29;11(11):1118. doi: 10.3390/jpm11111118 (PMC8621827; doi:10.3390/jpm11111118)
Supplement: Supplementary file 1 [file jpm-11-01118-s001.zip › jpm-1434355-supplementary.pdf]

SUPPLEMENTARY DATA

TABLE S1: Metadata (n=62) taken into account for correlation to overall taxon composition.

**Parameters**				
Gender				
Age				
Creatinine				
eGFR				
CKD_stage				
Diabetes				
Bristol Stool Scale				
Bacterial Cell counts				
Indoxyl sulfate (total)				
Indole Acetic Acid (total)				
p-cresyl sulfate/glucuronide (Total)				
Hippuric acid (total)				
**Medication in past 6 months (ATC-codes)**				
Alimentary tract and metabolisma	A05	A02	A09	A10
	A11	A12		
Blood and blood forming organs	B01	B03	B04	B05
Cardiovascular system	C01	C02	C03	C05
	C07	C08	C09	C10
Dermatological drugs	D01			
Genitourinary system and reproductive hormones	G02	G03	G04	
Systemic hormonal preparations, excluding reproductive hormones and insulins	HO2	H03	HG0	
Antiinfectives for systemic use	J01	J02	J05	J06
Antineoplastic and immunomodulating agents	L01	L02	L04	
Musculoskeletal system	M01	M04	M05	
Nervous system	N01	N02	N03	N04
	N05	N06	N07	
Antiparasitic products, insecticides and repellents	P01			
Respiratory system	R01	R03	R05	R06
Sensory organs	S01			
Various ATC structures	V01	V03		

eGFR: estimated glomerular filtration rate; CKD: chronic kidney disease; ATC: Anatomical Therapeutic Chemical (https://www.atccode.com/)

TABLE S2. Correlation between intestinally generated uremic toxins and transit time of patients with CKD not on laxatives.

**Correlation to BSS**	**All CKD stages (n = 101)**		**CKD stage 1-2 (n = 33)**		**CKD stage 3 (n = 39)**		**CKD stage 4-5 (n = 29)**	
**Uremic toxin**	r_s	**p-value**	r_s	**p-value**	r_s	**p-value**	r_s	**p-value**
**Indoxyl sulfate**	-0.162	ns	-0.162	ns	-0.010	ns	-0.276	ns
**Indole-3-acetic acid**	-0.107	ns	-0.060	ns	-0.021	ns	-0.162	ns
**Hippuric acid**	**-0.344**	**0.001**	**-0.420**	**0.021**	-0.224	ns	-0.313	ns
***p*-Cresyl sulfate**	**-0.271**	**0.008**	-0.359	ns	-0.200	ns	**-0.424**	**0.025**
***p*-Cresyl glucuronide**	-0.154	ns	-0.096	ns	-0.171	ns	-0.344	ns

BSS: Bristol stool scale which is used to assess transit time (slow transit = low BSS); r_s: Spearman's correlation coefficient; ns: not significant.
